# Variations in Unmet Health Care Needs by Perceptions of Social Media Health Mis- and Disinformation, Frequency of Social Media Use, Medical Trust, and Medical Care Discrimination: Cross-Sectional Study

**DOI:** 10.2196/56881

**Published:** 2024-07-11

**Authors:** Jim P Stimpson, Sungchul Park, Fernando A Wilson, Alexander N Ortega

**Affiliations:** 1 Peter O'Donnell Jr. School of Public Health, University of Texas Southwestern Medical Center, Dallas, TX, United States; 2 Department of Health Policy and Management, Korea University, Seol, Republic of Korea; 3 Matheson Center for Health Care Studies, University of Utah, Salt Lake City, UT, United States; 4 Thompson School of Social Work and Public Health, University of Hawaiʻi at Mānoa, Honolulu, HI, United States

**Keywords:** United States, cross-sectional study, trust, consumer health information, misinformation, disinformation, perceived discrimination, social media, unmet need, unmet needs, health care, discrimination, racism, adult, adults, medical care, frequency, multivariable regression, user, users, cross-sectional, survey, surveys, questionnaire, questionnaires, HINTS, Health Information National Trends Survey

## Abstract

**Background:**

Unmet need for health care is defined as choosing to postpone or completely avoid necessary medical treatment despite having a need for it, which can worsen current conditions or contribute to new health problems. The emerging infodemic can be a barrier that prevents people from accessing quality health information, contributing to lower levels of seeking medical care when needed.

**Objective:**

We evaluated the association between perceptions of health mis- and disinformation on social media and unmet need for health care. In addition, we evaluated mechanisms for this relationship, including frequency of social media use, medical trust, and medical care discrimination.

**Methods:**

Data from 3964 active adult social media users responding to the 2022 Health Information National Trends Survey 6 (HINTS 6), a nationally representative survey, were analyzed. The outcome was unmet need for medical care, defined as delaying or not getting the necessary medical care. The predictor variables were perception of social media health mis- and disinformation, frequency of social media use, level of trust in the health care system, and perceived racial and ethnic discrimination when receiving health care.

**Results:**

Multivariable logistic regression models indicated that perception of substantial social media health mis- and disinformation (odds ratio [OR] 1.40, 95% CI 1.07‐1.82), daily use of social media (OR 1.34, 95% CI 1.01‐1.79), low medical trust (OR 1.46, 95% CI 1.06‐2.01), and perceived discrimination (OR 2.24, 95% CI 1.44‐3.50) were significantly associated with a higher likelihood of unmet need for medical care. Unmet need among adults who did not use social media daily and who did not perceive substantial mis- and disinformation (24%; 95% CI 19%‐30%) was lower compared to daily social media users who perceived substantial mis- and disinformation (38%; 95% CI 32%‐43%). Adults who perceived substantial mis- and disinformation and had low trust in health care had the highest probability of reporting unmet need (43%; 95% CI 38%‐49%) compared to the other three groups. Adults who perceived substantial mis- and disinformation and experienced medical care discrimination had a statistically significant higher probability of reporting unmet need (51%; 95% CI 40%‐62%) compared to adults who did not experience medical care discrimination and did not perceive substantial mis- and disinformation (29%; 95% CI 26%‐32%).

**Conclusions:**

Unmet need for medical care was higher among individuals who perceived a substantial degree of social media mis- and disinformation, especially among those who used social media daily, did not trust the health care system, and experienced racial or ethnic discrimination when receiving health care. To counter the negative effects of social media mis- and disinformation on unmet need for health care, public health messaging must focus on daily social media users as well as improving trust and reducing structural racism in the health care system.

## Introduction

Unmet health care needs arise when someone with a known health issue skips or delays necessary medical treatments, such as rescheduling appointments, refusing treatments, or avoiding preventive measures [1-3]. This can happen for various reasons, including costs or difficulty accessing care [4]. Unfortunately, unmet health care needs can lead to untreated conditions becoming worse or developing new health problems. Certain populations are more likely to have unmet need for care, such as individuals with impairments, older people, marginalized racial and ethnic groups, and those without health insurance [5-8]. Systematic reviews have revealed areas that require further investigation [9,10]. For example, patient perceptions about the health care system and their own health conditions could be a significant factor contributing to unmet health care demands, underscoring a potential intervention option to improve unmet health care needs and subsequently improve health outcomes [7,9].

### Theoretical Framework

The health care utilization model developed by Anderson provides a strong foundation for analyzing the complex problem of unmet need for health care [11]. This model identifies several pathways that influence health care seeking and utilization, including resources that facilitate help-seeking, perceived and assessed need for care, and predisposing qualities. Social structures, health attitudes, and demographic traits are examples of predisposing characteristics that may influence an individual’s decision to use health services. The practical components of accessing care, such as the availability of health insurance, the accessibility of health care professionals, and socioeconomic position, are referred to as enabling resources. The choice to seek care is influenced by the patient’s perception of need and the assessment of medical professionals. Finally, contextual factors, such as public policies and the community environment, can influence each of these individual-level domains [7,11].

The way people receive, exchange, and act upon health information is greatly influenced by the communication infrastructure, which consists of networks, information technologies, and channels for both interpersonal and mediated communication [12]. In the Anderson model, the communication infrastructure can be viewed as a contextual factor that affects the predisposing qualities, enabling resources, and need factors. For instance, the information gleaned from many media sources may influence one’s opinions about health, thereby influencing the likelihood of seeking medical care [13,14].

The communication infrastructure also has an impact on enabling resources, which includes the practical aspects of receiving treatment. People can choose when and where to seek care by having access to trustworthy communication channels and accurate health information [14]. The quality and accessibility of health information has a direct bearing on the demand for health care, which motivates the actual use of services [12]. Unmet health care needs might arise from underestimating health needs or from mistrusting the health care system due to inaccurate or incomplete information [14]. Furthermore, poor communication can worsen gaps in access to health care, especially for racial and ethnic minorities who do not have easy access to reliable health information [15]. By integrating the communication infrastructure into the Anderson model, we can gain a deeper understanding of the complex relationship among health literacy, information availability, and health care use.

### Misinformation and Disinformation

The term “infodemic” describes the ongoing dissemination of false information and health-related falsehoods, and it has emerged as a significant public health issue [16]. Research has shown that the spread of misinformation, which refers to spreading inaccurate health information, and disinformation, which refers to intentionally disseminating false information, has had a negative effect on health-related behaviors and attitudes [17-21]. The dissemination of misinformation and disinformation via social media introduces a novel extension to the traditional Anderson model by representing a contextual factor influencing individual-level predisposing, enabling, and need factors. The frequency of social media use can either facilitate or hinder health care use, depending on the quality of information accessed and the user’s level of engagement [22-24]. Disseminating inaccurate information can undermine public confidence in the health care system, which is crucial for accessing health care services and can influence the decision to seek medical attention [25-28]. Moreover, the intentional dissemination of inaccurate information and misinformation to communities of color and ethnic minorities worsens preexisting disparities and perceptions of bias in the health care system, potentially leading to higher levels of unaddressed health care needs within these populations [20,29,30]. By integrating the communication infrastructure with the Anderson model, we can understand the communication barriers that prevent people from accessing health care and develop effective ways to mitigate the negative effects of health misinformation on public health [31].

### Mechanisms Linking Mis- and Disinformation and Health Care Use

The exposure to misinformation and disinformation on social media platforms can be connected to unmet health care requirements through various pathways, some of which have not been thoroughly studied in existing literature. The first mechanism pertains to the relationship between the use of social media and the level of engagement with material, specifically pertaining to false or misleading information [23]. Increased use of social media correlates with a higher probability of individuals interacting with different forms of content, including deceptive or inaccurate information [32]. Exposure to this digital information environment can modify a person’s beliefs and decision-making processes surrounding health care, potentially resulting in unmet need for medical care [33,34].

The second mechanism pertains to the confidence individuals place in health care institutions and professionals. Trust has a crucial role in influencing individuals’ decision to seek health care services and adhere to the treatments recommended by health care providers [35]. Certain groups are less likely to trust credible health institutions like government health agencies or health systems, but misinformation can influence the level of trust regardless of group membership [36-38]. Nevertheless, the spread of false information on social media undermines this trust, leading individuals to lose confidence in the health care institution and not seek medical attention when necessary [39-42].

The third mechanism emphasizes the problem of unmet need for care among racial and ethnic minority groups, which is frequently worsened by discrimination inside the health care system [43]. Social media platforms can serve as conduits for disseminating health-related disinformation, often aimed at these specific communities [44]. The deliberate dissemination of false information, specifically aimed at racial and ethnic minoritized populations, along with experiences of discrimination, can further erode trust in health care and result in higher levels of unmet health care needs [45-50]. These mechanisms collectively contribute to additional barriers in obtaining medical treatment. To effectively tackle the challenges presented by misinformation and its influence on public health, it is crucial to better understand how these mechanisms influence health care seeking and use.

### Research Objective

By integrating the communication infrastructure into the Anderson model of health care utilization, our study aimed to investigate the effects of the infodemic on the use of health care, proposing that exposure to health misinformation is associated with a higher probability of delaying or avoiding necessary medical care. In addition, our goal was to investigate the underlying mechanisms of this association, such as the frequency of social media use, the degree of trust that individuals have in the health care system, and the occurrence of racial or ethnic discrimination in health care settings. Our hypothesis is that people who perceive substantial false or misleading health information and use social media daily, have little trust in the health care system, and experience racial or ethnic discrimination when trying to use health care services are more likely to have unmet health care needs.

## Methods

### Data

This cross-sectional study analyzed nationally representative data from the Health Information National Trends Survey 6 (HINTS 6), which periodically surveys noninstitutionalized adults in the United States about information seeking, health communication, as well as cancer prevention attitudes and behaviors. The most recent version of the data, HINTS 6, were collected through mail- and web-based surveys from March to November 2022 with a response rate of 28.1% (34,827,468/124,058,843) [51].

### Ethical Considerations

The data were publicly available and deidentified; therefore, the human research protection program of the University of Texas Southwestern Medical Center decided it did not require review by the institutional review board. Further details about the survey methodology are available from the National Cancer Institute [51]. We excluded participants who did not report using social media or had not used social media in the past year. We analyzed data from 3964 adult social media users that had used social media in the past year, with complete data on the study variables.

### Measures

The binary outcome variable was unmet need for medical care, defined as delaying or not getting the medical care that the participant believed was medically necessary. The primary predictor variable was perceptions about health mis- and disinformation on social media, which was assessed by the following question: “How much of the health information that you see on social media do you think is false or misleading?” The response categories were dichotomized for ease of interpretation as substantial (ie, “a lot”) versus less than substantial (ie, “none, a little, some”), consistent with past studies [20,29,37]. The secondary predictor variables suggested by the theorectical framework included frequency of social media use, trust in the health care system, as well as perceived racial and ethnic discrimination [11-13]. Social media use was categorized as daily use versus less than daily use (eg, “never, weekly, monthly”). For medical trust, participants were asked “How much do you trust the health care system (for example, hospitals, pharmacies, and other organizations involved in health care)?” The response categories were dichotomized for ease of interpretation based on prior literature as high (“very” and “somewhat”) versus low (“not at all” and “a little”) [52,53]. For medical care discrimination, participants were asked “Have you ever been treated unfairly or been discriminated against when getting medical care because of your race or ethnicity?” and the response categories were “yes” or “no.”

The Andersen model for health care utilization was used to select the control variables [11]. Predisposing factors included age (18‐49, 50‐64, ≥65 years), sex (male and female), marital status (married or cohabiting, formerly married, and never married), self-reported race and ethnicity (non-Latino White, non-Latino Black, non-Latino other, and Latino), education level (college degree or higher vs less than a college degree), and residence in a metropolitan versus nonmetropolitan county as designated by the US Department of Agriculture in 2013. Enabling factors included full-time employment status, feelings about household income (finding it very difficult/difficult to get by on present income, or getting by on present income, living comfortably on present income), health insurance status (any insurance or uninsured), and frequency of health provider visits (0‐3 or more than 4 annual visits). The need for health care was measured by self-rated overall health status (excellent, very good, good, fair, and poor).

### Statistical Analysis

The descriptive statistics for the study sample were calculated as survey-weighted percentages. The bivariable relationships between the outcome and the predictor variables were calculated using column percentages and adjusted Wald *P* values. Then, we used a stepped multivariable logistic regression model to test for the main effects of perceptions of social media health mis- and disinformation on unmet health care needs. The first step adjusted for secondary predictor variables of social media use, medical trust, and medical care discrimination; the second model further adjusted for predisposing, enabling, and need factors [11]. In addition to the main effects, an interaction effect was calculated between the primary predictor variable and the secondary predictor variables to estimate the theorized pathways between mis- or disinformation on social media and unmet need for health care in multivariable logistic regression models. The interaction results were converted into predicted probabilities using the margins command in Stata (StataNow 18.5; StataCorp) for ease of interpretation. Sensitivity analyses were conducted that excluded non-Latino White individuals for the interaction effect of medical discrimination and perceived mis- or disinformation. All analyses accounted for survey weights and design using jackknife replicate weights for variance estimation. Statistical significance was set at α<.05.

## Results

Table 1 provides the unadjusted sample size and survey-weighted percentage points for all study variables among adult social media users in the past year. Most participants (n=2655, 67%) reported that their health care needs were met; a third of the participants (n=1309, 33%) reported unmet need for health care. More than a third of participants (n=1397, 36%) perceived substantial social media health mis- and disinformation. Most participants (n=2803, 73%) used social media daily. Low trust in the health care system was reported by 17% (n=596) of the participants, and 8% (n=348) reported experiencing racial or ethnic discrimination when seeking health care.

Table 2 shows the survey-weighted bivariable column percentages, showing statistically significant relationships between the outcome and predictors. Among participants who perceived substantial social media mis- and disinformation, 42% reported unmet need compared to 33% who reported their need for care was met. Among participants who reported daily social media use, 77% reported unmet need compared to 71% who reported their need for care was met. Among participants who reported low trust in the health care system, 24% reported unmet need compared to 14% who reported their need for care was met. Among participants who experienced racial or ethnic discrimination in receiving medical care, 13% reported unmet need compared to 5% who reported their need for care was met.

In Table 3, the first model of the multivariable logistic regression shows the association of the predictor variables without adjustment for covariates. Perception of substantial social media health mis- and disinformation (OR 1.41, 95% CI 1.09‐1.81), daily use of social media (OR 1.43, 95% CI 1.09‐1.87), low health care system trust (OR 1.74, 95% CI 1.29‐2.35), and perceived discrimination (OR 2.34, 95% CI 1.47‐3.73) were all associated with a higher likelihood of unmet need for medical care. In model 2, after adjusting for predisposing, enabling, and need factors, perception of substantial social media health mis- and disinformation (OR 1.40, 95% CI 1.07‐1.82), daily use of social media (OR 1.34, 95% CI 1.01‐1.79), low health care system trust (OR 1.46, 95% CI 1.06‐2.01), and perceived discrimination (OR 2.24, 95% CI 1.44‐3.50) remained significantly associated with a higher likelihood of unmet need for medical care.

Table 4 show the results from multivariable logistic models, adjusted for predisposing, enabling, and need factors, in which perception of mis- and disinformation was multiplied by predictors hypothesized to be mechanisms of the relationship between unmet need for care and perception of mis- and disinformation. There was a statistically significant difference in the probability of reporting unmet need for care among adults who did not use social media daily and who did not perceive substantial mis- and disinformation (24%; 95% CI 19%‐30%) compared to daily social media users who perceived substantial mis- and disinformation (38%; 95% CI 32%‐43%). Adults who perceived substantial mis- and disinformation and had low trust in the health care system had the highest probability of reporting unmet need for care (43%; 95% CI 38%‐49%) compared to the other three groups. Adults who perceived substantial mis- and disinformation and experienced medical care discrimination had a statistically significant higher probability of reporting unmet need for care (51%; 95% CI 40%‐62%) compared to adults who did not experience medical care discrimination and did not perceive substantial mis- and disinformation (29%; 95% CI 26%‐32%). We conducted a sensitivity analysis for the interaction of medical care discrimination and perception of mis- and disinformation, excluding non-Latino White participants, which resulted in a sample size of 1692. The results were replicated. Racially and ethnically minoritized adult social media users who perceived substantial mis- and disinformation and experienced medical care discrimination had a statistically significant higher probability of reporting unmet need for medical care (52%; 95% CI 38%‐67%) compared to racially and ethnically minoritized adult social media users who did not experience medical care discrimination and did not perceive substantial mis- and disinformation (24%; 95% CI 20%‐28%).

**Table 1.** Unadjusted sample size and survey-weighted percentages for study variables from the 2022 Health Information National Trends Survey 6 (N=3964).

Variable			Values, n (weighted %)	
**Outcome**				
	Unmet need for medical care (no)		2655 (67)	
	Unmet need for medical care (yes)		1309 (33)	
**Predictors**				
	**Perception of social media health mis- or disinformation**			
		<Substantial	2567 (64)	
		Substantial	1397 (36)	
	**Frequency of social media use**			
		<Daily	1161 (27)	
		Daily	2803 (73)	
	**Health care system trust**			
		High	3368 (83)	
		Low	596 (17)	
	**Medical care discrimination**			
		No	3616 (92)	
		Yes	348 (8)	
**Predisposing factors**				
	**Age group (years)**			
		18‐49	1751 (59)	
		50‐64	1185 (27)	
		≥65	1028 (14)	
	**Sex**			
		Male	1475 (47)	
		Female	2489 (53)	
	**Marital status**			
		Married or cohabiting	2208 (57)	
		Formerly married	928 (10)	
		Never married	828 (33)	
	**Rural and urban designation**			
		Nonmetro	489 (12)	
		Metro	3475 (88)	
	**Race and ethnicity**			
		Non-Latino White	2272 (61)	
		Non-Latino Black	613 (11)	
		Latino	725 (18)	
		Non-Latino other	354 (10)	
	**Education**			
		Not a college graduate	1915 (64)	
		College graduate or higher	2049 (36)	
**Enabling factors**				
	**Full-time employment**			
		No	1774 (40)	
		Yes	2190 (60)	
	**Feelings about household income**			
		Finding it very difficult/difficult to get by on present income	773 (19)	
		Getting by on present income	1453 (37)	
		Living comfortably on present income	1738 (44)	
	**Covered by any health insurance**			
		No	337 (11)	
		Yes	3627 (89)	
	**Number of health care provider annual visits**			
		0‐3	237 (65)	
		≥4	159 (35)	
**Need factors**				
	Fair or poor general health		634 (15)	
	Excellent, very good, or good general health		3330 (85)	

**Table 2.** Survey-weighted bivariable column percentages for unmet medical care needs, perception of health mis- and disinformation on social media, frequency of social media use, trust in the health care system, and experience of racial or ethnic discrimination when receiving medical care among adult social media users in the past year from the 2022 Health Information National Trends Survey 6 (N=3964).

Variable		Met need for medical care, weighted %	Unmet need for medical care, weighted %	*P* value^a^
**Perceptions of social media health mis- and disinformation**				.01
	<Substantial	67	58	
	Substantial	33	42	
**Frequency of social media use**				.02
	<Daily	29	23	
	Daily	71	77	
**Health care system trust**				<.001
	High	86	76	
	Low	14	24	
**Medical care discrimination**				<.001
	No	95	87	
	Yes	5	13	

a *P* values were calculated using the adjusted Wald *χ*2 test.

**Table 3.** Multivariable odds ratios (ORs) and 95% CIs for unmet need for medical care among social media users in the past year derived from the 2022 Health Information National Trends Survey 6 (N=3964). Logistic regression models were adjusted for survey weight and design. Model 1 shows the association of the predictor variables without adjustment for covariates.Model 2 added adjustments for age, sex, marital status, urban or rural designation, race and ethnicity, education, employment status, feelings about household income, health insurance coverage, number of health care provider visits, and general health status.

Variable		Model 1, OR (95% CI)	Model 2, OR (95% CI)
**Perception of social media health mis- and disinformation**			
	<Substantial	Reference	Reference
	Substantial	1.41 (1.09‐1.81)	1.40 (1.07‐1.82)
**Frequency of social media use**			
	<Daily	Reference	Reference
	Daily	1.43 (1.09‐1.87)	1.34 (1.01‐1.79)
**Health care system trust**			
	High	Reference	Reference
	Low	1.74 (1.29‐2.35)	1.46 (1.06‐2.01)
**Medical care discrimination**			
	No	Reference	Reference
	Yes	2.34 (1.47‐3.73)	2.24 (1.44‐3.50)

**Table 4.** Multivariable-adjusted percentage points for unmet need for medical care and the interaction effect between perceptions of health mis- and disinformation on social media and frequency of social media use, health care system trust, and medical care discrimination from the 2022 Health Information National Trends Survey 6 (N=3964). Predicted marginal effects were calculated from multivariable logistic regression models that were adjusted for survey weight and design, age, gender, marital status, urban or rural designation, race and ethnicity, education, employment status, feelings about household income, health insurance coverage, number of health care provider visits, and general health status.

Variable		Perceptions of health mis- and disinformation on social media, % (95% CI)	
		<Substantial	Substantial
**Frequency of social media use**			
	<Daily	24 (19‐30)	36 (28‐44)
	Daily	33 (28‐37)	38 (32‐43)
**Health care system trust**			
	High	30 (24‐36)	25 (18‐32)
	Low	31 (27‐34)	43 (38‐49)
**Medical care discrimination**			
	No	29 (26‐32)	36 (31‐41)
	Yes	49 (37‐61)	51 (40‐62)

## Discussion

### Principal Findings

The primary objective of this study was to apply an extension of the Anderson model of health care utilization by evaluating the communication environment as a contextual determinant of health care seeking and utilization, which had not been specifically explored in prior studies [11-13]. Toward this end, we examined the association between perceptions of health mis- and disinformation on social media and unmet need for health care. We found that perception of substantial social media mis- and disinformation was associated with a higher probability of reporting unmet health care need compared to those who did not perceive substantial mis- and disinformation. This result is consistent with a growing body of literature demonstrating the negative impacts of social media health mis- and disinformation on health outcomes [16-19]. Our findings suggest that the communication environment serves as a contextual determinant of seeking health care by exposing individuals to false health information that is then associated with people delaying or avoiding medical care, even when it is needed, resulting in worse health outcomes [14]. Thus, perceptions of substantial health mis- and disinformation on social media may serve as one possible underlying mechanism for unmet need for health care.

In addition, we evaluated whether the relationship between perceptions of health mis- and disinformation on social media and unmet need for health care depended on the frequency of social media use, trust in the health care system, and experience of racial and ethnic discrimination in health care. We found that unmet need for medical care was lower among adults who did not use social media daily and did not perceive substantial mis- and disinformation compared to daily social media users who perceived substantial mis- and disinformation. This finding is consistent with our theoretical framework that integrated the Anderson model of health care utilization with the frameoworks that view the communication environment as a social driver of health [11,12]. Our empirical findings and theoretical framework suggest that more time spent on social media increases the exposure to and engagement with mis- and disinformation, which can lead frequent daily social media users to be more likely to have false beliefs that lower their likelihood of seeking needed medical care [32-34].

For the second mechanism, adults with low trust in the health care system and those who perceive substantial mis- and disinformation had the highest probability of reporting unmet health needs. This finding is consistent with prior literature and our theoretical framework demonstrating a trend toward eroding trust in medical professionals and health institutions associated with social media health mis- and disinformation, which influences harmful health behaviors and poor health outcomes [36-42]. Trust in the health care system is important for patient compliance with recommended care and seeking necessary medical care, and trust is difficult to rebuild once lost, suggesting this mechanism is critical for reducing negative impacts of unmet need for medical care [4,35].

Finally, adults who perceived substantial mis- and disinformation and experienced medical care discrimination had a higher probability of reporting unmet need for medical care compared to adults who did not experience medical care discrimination and did not perceive substantial mis- and disinformation. This finding is consistent with a large body of literature and our theoretical framework demonstrating the negative impacts of racial and ethnic discrimination experiences for seeking needed health care [43-50]. Our results and recent studies suggest that experiencing discrimination may have a complex relationship with social media health mis- and disinformation by further reducing historical levels of low trust in health care institutions [43,52,53].

### Limitations

There are several limitations to consider when interpreting the results. First, a nonresponse analysis of a prior iteration of HINTS from 2011 and 2013 suggested that estimates using measures on seeking health information may be higher than other surveys [54]. To mitigate this potential effect and increase the validity of the study results, we excluded individuals who did not use social media and those who had not visited a social media platform in the past year (during the study period). Of course, the findings are the result of a cross-sectional survey, and therefore, should not be interpreted to indicate a causal relationship. One final context for interpreting the findings is that this study was focused on social media health mis- and disinformation and the findings might not apply to mis- and disinformation in other health contexts.

### Conclusions

Based on our expanded version of the Anderson health care utilization model, we found that unmet need for health care was higher among individuals who perceived a substantial amount of social media mis- and disinformation, especially among those who used social media daily, had a low trust in the health care system, and experienced racial or ethnic discrimination when receiving health care. Our findings justify an expansion of the Anderson model of health care utilization by conceptualizing the communication environment as a contextual determinant of seeking health care. Future research should continue to model the communication environment as a contextual determinant of health care utilization and further evaluate the mechanisms of exposure to health mis- and disinformation, such as trust and discrimination. By extension, another area of future research could be the role of digital literacy, which we did not measure in this study. Individuals possessing advanced digital literacy skills are more likely to have the ability to differentiate between reliable health information and misleading or false information, and therefore, digital literacy may influence the connection between social media use and unmet health care needs [55]. Another area of future research is the role that social media plays in the support networks for individuals with specific health issues, perhaps mitigating or exacerbating the adverse impacts of mis- and disinformation through peer-supplied information and emotional support, which could interact with trust and past experiences of discrimination [55].

To counter the negative effects of social media mis- and disinformation on unmet need for health care, tailored public health efforts on social media are needed. These efforts should specifically target prevailing misunderstandings and offer fact-based information that has the potential to diminish the spread and consequences of health mis- and disinformation. As a component of this effort, health care institutions could actively monitor social media patterns to swiftly detect and address the emergence of health-related false and misleading information, potentially in real-time [55,56]. By implementing patient education programs that focus on developing health literacy, especially in the assessment of web-based health information, patients could be empowered to make well-informed health care choices [57]. Finally, health care institutions should continue to work toward building trust and reducing structural racism to mitigate the effects of mis- and disinformation and encourage patients to access health services when needed.

## Abbreviations

HINTS: Health Information National Trends Survey
OR: odds ratio
